# Insulin and insulin-like growth factor-I receptors in astrocytes exert different effects on behavior and Alzheimer´s-like pathology

**DOI:** 10.12688/f1000research.121901.3

**Published:** 2022-12-20

**Authors:** Jonathan Zegarra-Valdivia, Ana M. Fernandez, Laura Martinez-Rachadell, Raquel Herrero-Labrador, Jansen Fernandes, Ignacio Torres Aleman

**Affiliations:** 1 Achucarro Basque Center for Neuroscience, Leioa, Bizkaia, 48940, Spain; 2 Cajal Institute, Madrid, 28002, Spain; 3 CIBERNED, Madrid, Spain; 4 Universidad Señor de Sipán, Chiclayo, Peru; 5 Universidade Federal São Paulo, São Paulo, Brazil; 6 Ikerbasque Foundation for Science, Bilbao, Spain

**Keywords:** Astrocytes, Alzheimer`s disease, Insulin, Insulin-like growth factor I, Glial cells.

## Abstract

**Background: **Pleiotropic actions of insulin and insulin-like growth factor I (IGF-I) in the brain are context- and cell-dependent, but whether this holds for their receptors (insulin receptor (IR) and IGF-I receptor (IGF-IR), respectively), is less clear.

**Methods: **We compared mice lacking IR or IGF-IR in glial fibrillary astrocytic protein (GFAP)-expressing astrocytes in a tamoxifen-regulated manner, to clarify their role in this type of glial cells, as the majority of data of their actions in brain have been obtained in neurons.

**Results: **We observed that mice lacking IR in GFAP astrocytes (GFAP IR KO mice) develop mood disturbances and maintained intact cognition, while at the same time show greater pathology when cross-bred with APP/PS1 mice, a model of familial Alzheimer´s disease (AD). Conversely, mice lacking IGF-IR in GFAP astrocytes (GFAP-IGF-IR KO mice) show cognitive disturbances, maintained mood tone, and show control-dependent changes in AD-like pathology.

**Conclusions: **These observations confirm that the role of IR and IGF-IR in the brain is cell-specific and context-dependent.

## Introduction

Work in invertebrate insulin-like peptide (ILP) receptors, that recognize multiple ILP ligands ( Kimura *et al*., 1997), have provided valuable information on their pleiotropy ( Fernandes de Abreu *et al*., 2014). However, the vertebrate tyrosine kinase ILP receptors (IR) and IGF-IR specifically recognize insulin and IGF-I, respectively ( McGaugh *et al*., 2015), making it difficult to infer their role from observations gathered in invertebrate models. For instance, *daf-2*, the worm ILP receptor ( Kimura *et al*., 1997), interferes with mechanisms of proteostasis ( Cohen *et al*., 2006) and longevity ( Kenyon *et al*., 1993), whereas in vertebrates these roles has been tentatively assigned to IGF-IR ( Cohen *et al*., 2009) since the role of IR in these contexts is not yet clear ( Freude *et al*., 2009a; Shimizu *et al*., 2011). Moreover, the numerous actions of ILPs in physiology and pathology are context- and cell-dependent, which means that observations of the actions of ILPs in a given tissue or organ must be nuanced by the experimental approach used in each case. In brain studies, most of the information gathered on the role of IR and IGF-IR has been obtained after manipulating its function either in neurons ( Gontier *et al*., 2015; De Magalhaes Filho *et al*., 2016) or in many brain cell types at the same time ( Cohen *et al*., 2009; Soto *et al*., 2019).

Since recently published work shows that IR and IGF-IR in astrocytes play cell-dependent actions ( Cai *et al*., 2018; Noriega-Prieto *et al*., 2021), hinting to differential roles of these receptors in astrocytes, we compared behavioral traits in mice lacking IR in astrocytes with mice lacking IGF-IR in this type of cells. Mice with reduced IR in glial fibrillary astrocytic protein (GFAP) astrocytes (GFAP IR KO mice) show gradual mood disturbances and preserved cognition while mice with reduced IGF-IR in GFAP astrocytes (GFAP IGF-IR KO mice) show preserved mood and altered cognition. We also bred these mice in an APP/PS1 background mimicking familial AD-like amyloidosis and observed that GFAP IR KO mice develop significantly greater pathology whereas GFAP IGF-IR KO mice did not.

## Methods

Experimental models used in this study aimed to mimic human physio-pathology in relation to the established brain insulin and IGF-I resistance during healthy aging or AD. No protocol of these studies was prepared in advance.

### Animals

Mice were used according to Animal Research: Reporting of in vivo Experiments (ARRIVE) and this study is reported in line with the guidelines ( Zegarra-Valdivia *et al*., 2022). Transgenic mice with tamoxifen-regulated deletion of IGF-IR or IR in astrocytes (GFAP-IGF-IR KO and GFAP-IR KO mice, respectively) were obtained as described ( Garcia-Caceres *et al*., 2016; Noriega-Prieto *et al*., 2021) crossing IR ^f/f^ (B6.129S4(FVB)-Insr ^tm1Khn^/J RRID:IMSR, Jackson labs; stock number 006955) or IGF-IR ^f/f^ (B6, 129 background; Jackson Labs; stock number: 012251) with hGFAP-CreER ^T2^ mice (C57B&/6xSJL/J mix background Jackson Labs, stock number: 012849). To knock down the target gene, tamoxifen was administered to 2- months old mice for 5 days (75 mg/kg, Sigma, intraperitoneally) as described ( Hirrlinger *et al*., 2006), and animals were used one month later. Controls littermates received the vehicle (corn oil). GFAP-IGF-IR KO and GFAP-IR KO display reduced mRNA levels in brain, as reported by Noriega-Prieto *et al.* (2021) and Garcia-Caceres *et al.* (2016). GFAP-IR KO mice show brain IGF-IR levels similar to wild type mice whereas GFAP-IGF-IR KO mice had normal brain IR levels ( Hernandez-Garzon *et al.*, 2016). APP _swe_ and PS1Δ9 mice of C57BL6/J background were from the colony of the Cajal Institute. Homozygous APP/PS1 mice were crossed with homozygous GFAP IGF-IR KO or GFAP IR KO mice to obtain the respective compound strains. Studies were carried out at the age of 10-11 months-old, when pathology is well developed.

### Ethical considerations

Mice were were housed in standard cages (48 × 26 cm ^2^) with 5 mice per cage. Mice were maintained on a light-dark cycle (12-12 h, lights on at 8 am) at constant temperature (22°C) and humidity, and with food (pellet rodent diet) and water *ad libitum.* All experimental protocols were performed during the light cycle and followed European guidelines (86/609/EEC & 2003/65/EC, European Council Directives).

Studies were approved by the respective local Bioethics Committees (Government of the Community of Madrid, MERGEFIELD CÓDIGO PROEX 193.4/20 (2020) and UPV M20_2021_168 (2021). Animals were not randomized and were used in a sex-balanced manner throughout. Potential confounders were not accounted for. Each experimenter took account of group allocation under study. All efforts were done to reduce harm to the animals. Mice were handled for three days prior to any experimental manipulations and familiarized with behavioral arenas to minimize novelty stress or deeply anesthesized with pentobarbital prior to sacrifice, when needed. Sample sizes were kept as little as possible to comply with current animal reduction policies. No adverse events were expected, nor found. End-point measures included checking reflexes in deeply anesthesized animals prior to culling.

### Behavioral tests

These tests were used to determine behavior under laboratory-controlled conditions. These are observational studies with no *a priori* hypothesis.

*Barnes maze.* To assess spatial learning and memory, animals received reinforcement to escape from an open circular platform (92 cm **Ø** with 20 holes of 5 cm **Ø**) to the “escape chamber”, as described ( Ortiz *et al*., 2010; Zegarra-Valdivia *et al*., 2019). All animals received appropriate training (four trials per day), and trials were separated by 15 min. After each trial, the maze was cleaned with 70% alcohol. On the 5 ^th^ day, both groups were tested, and once more 48 hours later, evaluating the long-term memory of the animals. Time to escape to the safe chamber was quantified.

*Open field.* Exploratory behavior and locomotion were assessed by introducing the animal to an open field arena (42 cm × 42 cm × 30 cm, Versamax; AccuScan Instruments, Inc.) for 10 min. All parameters were quantified as described ( Zegarra-Valdivia *et al*., 2019). Time spent exploring specific areas of the arena was measured.

*Elevated plus maze.* To assess anxiety-like/coping behavior, mice were introduced in a maze of 40 cm from the floor with two opposing arms. Two protected (closed) arms (30 cm (length) × 5 cm (wide) × 15.25 (height), and two opposing unprotected (open) arms (30 cm (length) × 5 cm (wide). Each animal was introduced in the middle of the apparatus for 5 minutes. Stress was scored as time spent in the closed arms while coping behavior was estimated by time spent in the open arms. All measures were recorded (Video Tracking Plus Maze Mouse; Med Associates, USA), and analyzed as described ( Munive *et al*., 2019).

*Y-maze.* This test measures spontaneous alternation as an index of working memory ( Sarter *et al*., 1988). The maze is made of black-painted wood, and each arm is 25 cm long, 14 cm high, 5 cm wide, and positioned at equal angles. The mouse is placed at the end of one arm to move freely from side to side of the maze during an 8-min session. Videos recorded the sequence of entries during the whole time of the experiment and were analysed off-line. Entrance to each arm is scored when the mouse places the hind paws entirely in the zone. Alternation was defined as successive entries into the three arms on overlapping triplet sets. Consecutive triplets were analyzed, and alternate behaviour was calculated as the percentage of actual alternation (number of triplets with non-repeated entries) versus total alternation opportunities (total number of triplets), as described ( Recinto *et al*., 2012; Yan *et al*., 2017).

*Tail suspension.* In this test coping behaviors are determined. As already described ( Munive *et al.*, 2019), mice were suspended by the tail from a plastic cage (21×26×15) with adhesive tape (distance from tip of tail was 2 cm); the distance from the floor was 35 cm. Animals struggled to get to the floor until they give up and struggled less frequently. A 6 min test session was videotaped and time spent immobile was scored and referred as percent of total time of duration of the test.

*Forced swim.* This test measures depressive-like behavior. As described ( Munive *et al.*, 2019), mice were placed in a glass cylinder (12 cm diameter, 29 cm height) filled with water (23°C) to a height of 15 cm (to avoid climbing) and videotaped. The test lasted 6 min, and immobility time was scored the last 4 minutes.

*Spatial Y-maze.* This test was used for spatial, novelty-seeking, and short-term memory assessment by measuring time spent in the novel arm ( Hausrat *et al*., 2015; Biundo *et al*., 2018). As before, the maze was made of black-painted wood and each arm was 25 cm long, 14 cm high, 5 cm wide and positioned at equal angles. Each mouse was allowed to explore two arms of the Y-maze apparatus during the first trial (training) for 5 min. One hour later, the third arm was opened, and the mouse was returned to the same maze and allowed to explore all the three arms (testing). Visual cues were used to guide environment exploration, as described ( Biundo *et al*., 2018). Animals with preserved cognition remember the previously blocked arm and they will enter it first on the second trial and spend more time exploring it. Distribution of mice and novel arms were balanced within each group. We cleaned the maze with 70% ethanol to remove olfactory cues between trials.

*Rota-rod.* Motor coordination was assessed with the rota-rod test, as described before ( Fernandez *et al*., 1998). Briefly, mice were submitted to 1 min training session in the immobile apparatus. When the mouse falls, it is placed back into the rotating rod. Thereafter, mouse performance was tested in 5 min sessions every 15 min in 4 trials with increasing acceleration up to 40 rpm. The latency to fall off the rod in the final trial was measured and compared between groups.

*Social behavior.* Social behavior includes rewarding and motivational processes ( Trezza *et al.*, 2011; McCall and Singer, 2012). We studied social affiliation and social novelty/preference as described by others ( Kaidanovich-Beilin *et al.*, 2011). We placed each mouse in a cage with three compartments (one central and two lateral arms); in each compartment, we added a grid with one stranger mouse or an empty grid to assess social affiliation (intention to stay with the same species). We leave the mouse to explore for 10 minutes and record the time of direct interaction. Then, we cleaned the three chambers with ethanol (70%) to eliminate olfactory cues and placed the mice again in the center chamber. We include the previous stranger mice in the same arm (now named “familiar mouse”). In the empty space we include a new mouse (“stranger mouse”) and leave the animal free to explore and record the time of direct interaction.

### Immunocytochemistry

Immunocytochemistry was performed as described in detail before ( Fernandez *et al*., 2012). A pre-treatment of 70% formic acid was used before incubation with anti-human Aβ antibody (1:50, Dako clone 6F/3D). Primary antibody was omitted as control. Confocal analysis was performed in a Leica (SP5 Direct, Germany) microscope. For plaque morphometry, 1-4 vibratome brain sections (50 μm, parietal cortex and hippocampus) were used to assess the density of Aβ plaques using Imaris software (Vs 9.0.2) (RRID:SCR_007370). Measurements were done as explained in detail elsewhere ( Fernandez *et al*., 2012). Briefly, images were recorded using a 5X objective and were converted to gray scale to improve the contrast between signal and noise. All pictures were measured separately applying the same threshold. Areas were measured inside a reference circle in the hippocampus or parietal cortex with a standard size of 300 mm ^2^. We then calculated the percentage of reference area occupied by Aβ plaques.

### Statistics

The number of animals for each experiment was calculated according to past experience with no hypothesis-driven outcomes, as these are observational studies. All animals in each group were included in analyses with no exclusion criteria applied *a priori.* Values were relativized compared to the control or baseline condition. Results are expressed as the average of the relative values obtained in each independent test (mean ± standard error) for each experiment and analyzed with GraphPad Prism 8.0 software (RRID:SCR_002798) (alternative open access program: R Program). Normality was confirmed using the Shapiro-Wilk normality test and equal variances with Levene’s test. Later, student’s t-test was used for comparison of two groups, or ANOVA for comparison of more than two groups with a Tukey or Sidak’s *post-hoc* analysis. Further details are explained in each figure. A statistically significant difference was considered when p<0.05.

## Results

### Behavioral traits in mice lacking insulin or IGF-I receptors in astrocytes

**Figure 1.**
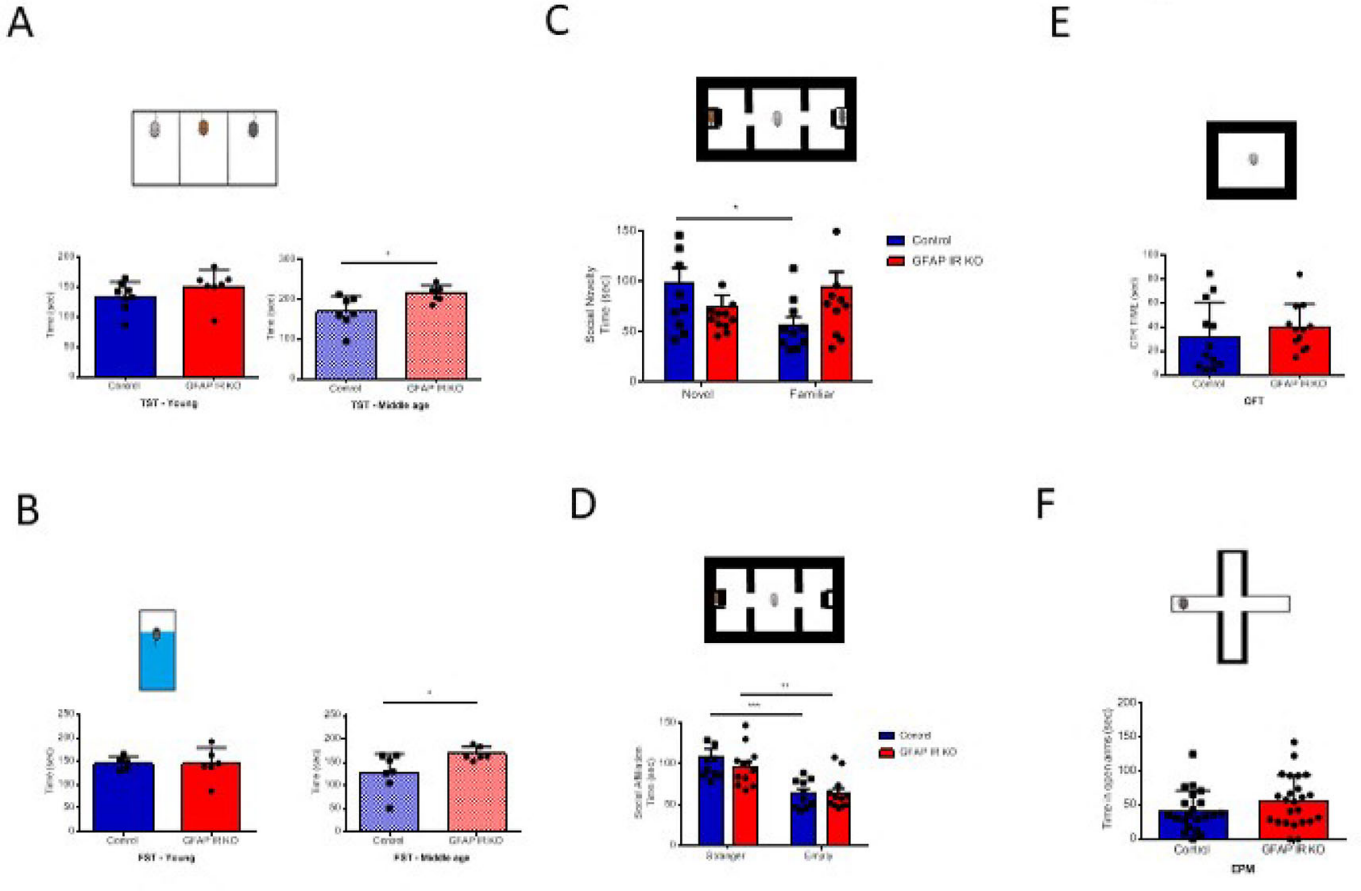
Mood traits in mice lacking insulin receptors (IR) in GFAP astrocytes (GFAP-IR KO mice). A, Adult (right histograms, control n= 6, GFAP IR KO n=7, t-test, t= 2.54; *p<0.05), but not young (left histograms, control n= 8, GFAP IR KO n=7, t-test, t= 1.26, p=0.22) GFAP IR KO mice show increased immobilization time in the tail suspension test (upper drawing), an indicator of a depressive-like behavior and reduced resilience to stress. B, Similarly, in the forced swim test (upper drawing), adult (right histograms, control n= 5, GFAP IR KO n=6, t-test, t= 2.5; *p<0.05, Welch`s correction), but not young (left histograms, control n= 5, GFAP IR KO n=6, t-test, t= 0.10; p=0.922) GFAP IR KO mice show increased depressive-like performance, with less time spent swimming. C, Social novelty, as measured by time spent with a novel partner vs a familiar one (upper drawing), was impaired in GFAP IR KO mice (control n= 10, GFAP IR KO n=12, t-test, t= 2.25; *p<0.05). D, Social affiliation, as determined by time spent with a stranger mouse vs an empty cage, was normal in GFAP IR KO mice (control n= 10, GFAP IR KO n=12, 2-way RM ANOVA, condition factor, F(1,20)=28.74; ***p<0.001, Sidak's multiple comparisons test, control familiar mice vs empty cage, ***p<0.001, GFAP IR KO novel vs familiar, **p<0.01). E, Time spent in the center of an open arena (upper drawing), a measure of novelty stress indicating levels of anxiety remained within control levels in adult GFAP IR KO mice (n=12 per group; t-test; t=0.77, p=0.445). F, Anxiety levels, as determined by time in the open arms of the elevated plus maze (upper drawing), are slightly, were normal in adult GFAP IR KO mice (control n= 21, GFAP IR KO n=24, t-test, t= 1.46, p=0.15). GFAP=glial fibrillary astrocytic protein.

**Figure 2.**
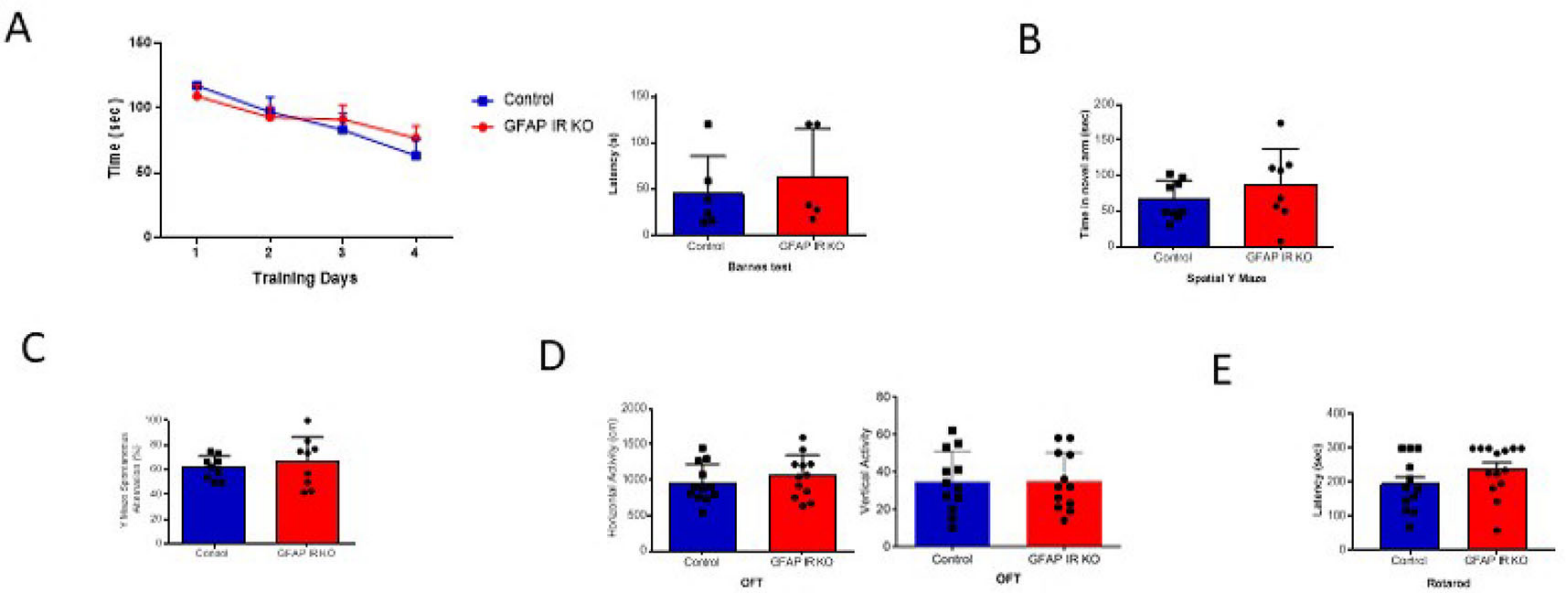
Cognition in GFAP-IR KO mice. A, Adult GFAP IR KO mice performed similarly in the Barnes maze as compared to littermates, indicating intact spatial learning (n=9 per group, training days: 2-way ANOVA: F(1,36)=0.10, p=0.74; test day: t-test, Welch’s correction, t=0.65, p=0.53). B, Time spent in the novel arm of the spatial Y maze was similar to littermates in GFAP IR KO mice (n=9 per group, t-test, t=1.24, p=0.23). C, Number of spontaneous alternations in the arms of the Spontaneous Alternation Y maze, a measure was similarly unaltered in adult GFAP IR KO mice (n=9 per group, t-test, Welch’s correction, t= 0.67, p=0.51). D, No differences were observed in horizontal (left histograms) and vertical (right) activity in the open field arena was observed between experimental groups (n=12 per group, H: t-test, t=0.84, p=0.40; V: t-test, t= 0.05, p=0.95). E, Control littermates and GFAP IR KO mice show similar levels of motor coordination, as assessed in the rota-rod (control n=12, GFAP IR KO n=14, H: t-test, t=1.52, p=0.14). IR=insulin receptors, GFAP=glial fibrillary astrocytic protein.

Recent publications in different models of downregulation of either insulin or IGF-I receptors in astrocytes have started to unveil specific actions of these receptors in this type of glial cells ( Cai *et al*., 2018; Logan *et al*., 2018; Manaserh *et al*., 2019; Noriega-Prieto *et al*., 2021). We confirm that adult GFAP IR KO mice gradually show a depressive-like phenotype ( Cai *et al*., 2018), as determined by the forced swim and the tail suspension tests. These alterations are seen in adult (>6 months old), but not younger mice ( Figure 1A- B) ( Zegarra-Valdivia *et al*., 2022). The existence of a depressive-like phenotype was reinforced by the observation that adult GFAP IR KO mice show disturbed responses to social novelty ( Figure 1C), although not to social affiliation ( Figure 1D). As determined in the open field test and elevated plus maze, GFAP IR KO mice did not show changes in anxiety levels either, which are frequently associated to depression ( Figure 1E- F). These mice have intact cognition, as determined in the Barnes and Y maze tests assessing learning and memory ( Figure 2A- C). In addition, GFAP IR KO mice did not show deficits in ambulation or motor coordination ( Figure 2C- D).

**Figure 3.**
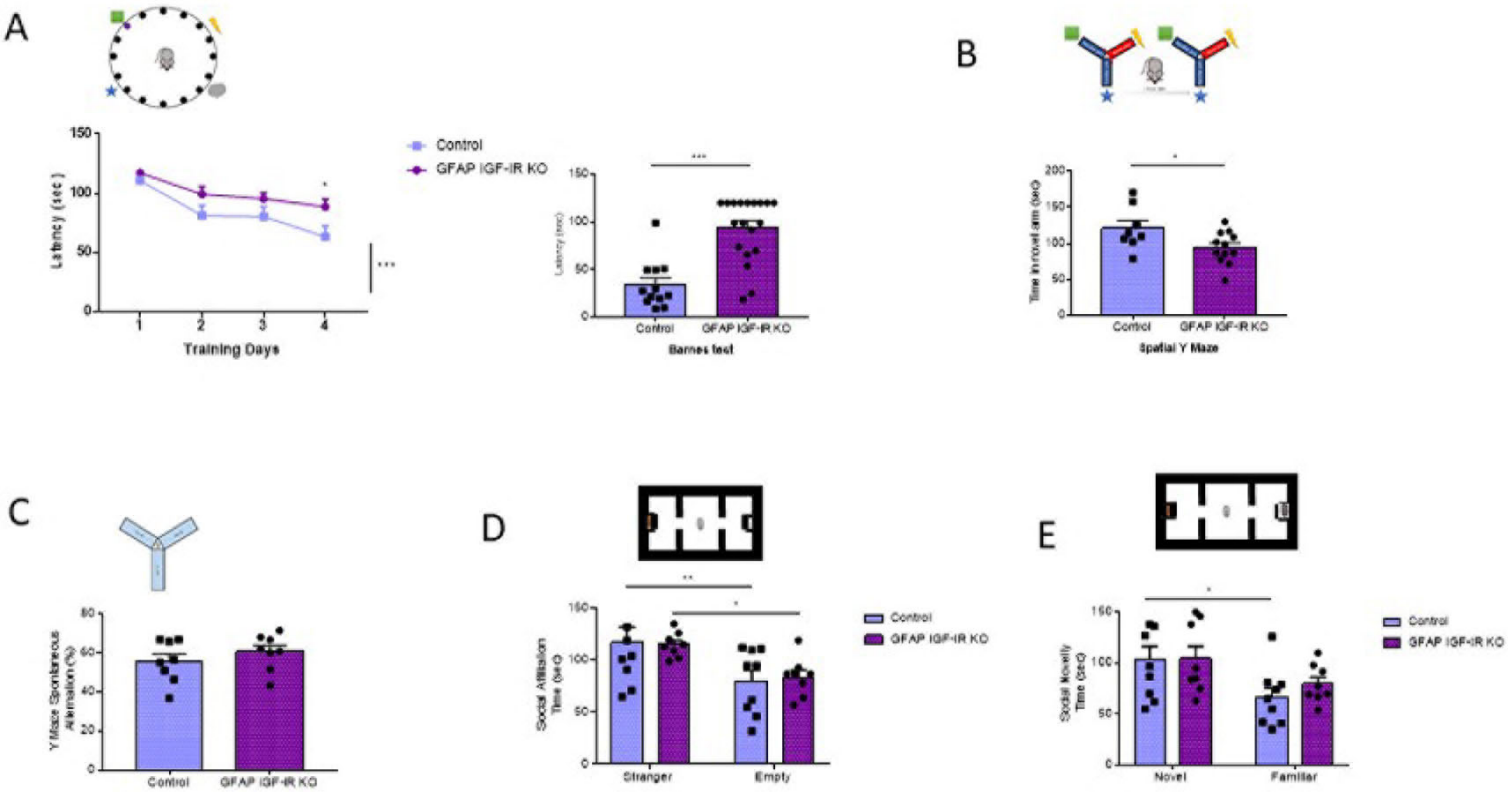
Cognitive traits in mice lacking IGF-I receptors (IGF-IR) in GFAP astrocytes (GFAP-IGF-IR KO mice). A, Spatial learning in the Barnes maze (upper drawing) was markedly affected in GFAP IGF-IR KO mice, showing significantly reduced memory (control n= 12, GFAP IR KO n=19, training days: 2-way ANOVA, time factor, F(3,122)=12.7; ***p<0.001, Sidak's multiple comparisons test, control vs GFAP 4 ^th^ day of training, *p<0.05; test day: Mann-Whitney U: 21.5, ***p<0.001). B, Time spent in the novel arm of the Y maze (upper drawing), a measure of spatial memory, was reduced in GFAP IGF-IR KO mice (control n= 8, GFAP IGF-IR KO n=12, t-test, t= 2.26, *p<0.05). C, Number of spontaneous alternations in a Y maze, a measure of working memory (upper drawing), was similarly unaltered in adult GFAP IGF-IR KO mice (n=8 per group; t-test; t=0.98, p=0.342). D, Social affiliation, as determined by time spent with a stranger mouse vs an empty cage (upper drawing), was normal in GFAP IGF-IR KO mice (control n=9, GFAP IGF-IR KO n=8, 2-way RM ANOVA, condition factor, F(1,15)=19.13; ***p<0.001, Sidak's multiple comparisons test, control familiar mice vs empty cage, **p<0.01, GFAP IR KO familiar vs empty cage, *p<0.05). E, Social novelty, as measured by time spent with a novel partner (upper drawing), was impaired in GFAP IGF-IR KO mice (control n=9, GFAP IGF-IR KO n=8, 2-way RM ANOVA, condition factor, F(1,15)=11.18; **p<0.01, Sidak's multiple comparisons test, control novel mice vs familiar mice, *p<0.05, GFAP IGF-IR KO novel mice vs familiar mice, p=0.16). GFAP=glial fibrillary astrocytic protein.

**Figure 4.**
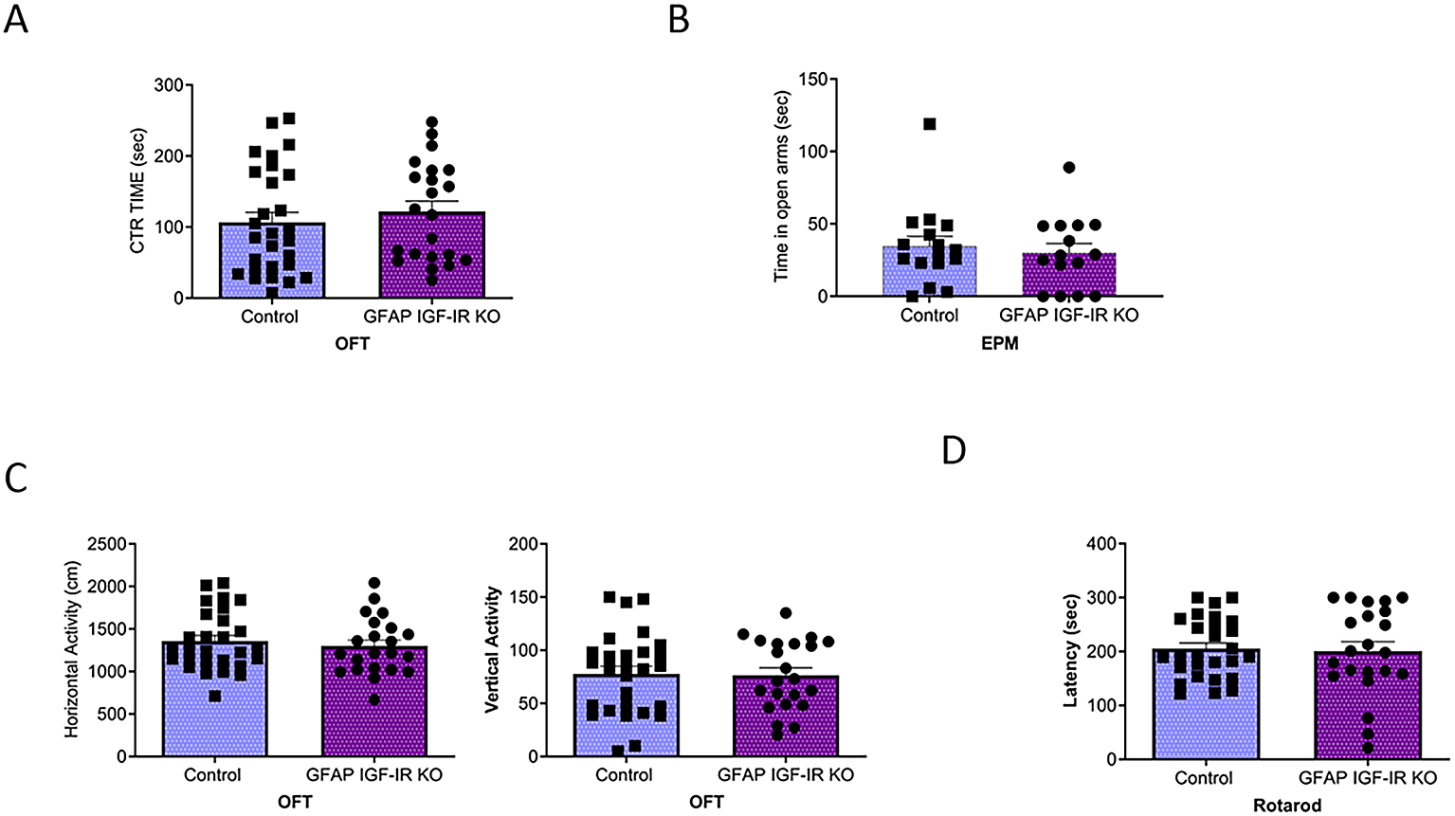
Mood homeostasis in GFAP IGF-IR KO mice. A, No differences in time spent in the center of an open field were observed between littermates and mutant GFAP IGF-IR KO mice (control n=28, GFAP IGF-IR KO n=22, t-test, t=0.73, p=0.46). B, No differences were observed in anxiety levels determined in the EPM between GFAP-IGF-IR KO and littermates (control n=16, GFAP IGF-IR KO n=15, t-test, t=0.48, p=0.63). C, No differences were observed in horizontal (left histograms) and vertical (right) activity in the open field arena was observed between GFAP IGF-IR KO mice and littermates (control n=28, GFAP IR KO n=22, H: t-test, t=0.61, p=0.54; V: t-test, t=0.12, p=0.90). D, Control and GFAP-IGF-IR KO mice show similar levels of motor coordination, as assessed in the rota-rod (control n=22, GFAP IR KO n=27, t-test, t=0.22, p=0.82).

Conversely, adult (>6 months old) GFAP IGF-IR KO mice show specific impairments in spatial memory as assessed in the Barnes and Y mazes ( Figure 3A- B), confirming previously observed deficits in cognition in these mice ( Noriega-Prieto *et al*., 2021) *.* However, working memory, as assessed by the alternation ratio in the Y maze, was intact ( Figure 3C). These mice show normal social affiliation, whereas their preference for a novel partner was slightly impaired ( Figure 3D- E). GFAP IGF-IR KO mice did not show mood disturbances either, as determined by time spent in the center of an open arena or in the open arms of the elevated plus maze ( Figure 4A- B). GFAP IGF-IR KO show normal ambulatory behavior in the open field ( Figure 4C), and in motor coordination tested in the rota-rod ( Figure 4D).

### Modulation of Alzheimer’s-like pathology in mice lacking insulin or IGF-I receptors in astrocytes

**Figure 5.**
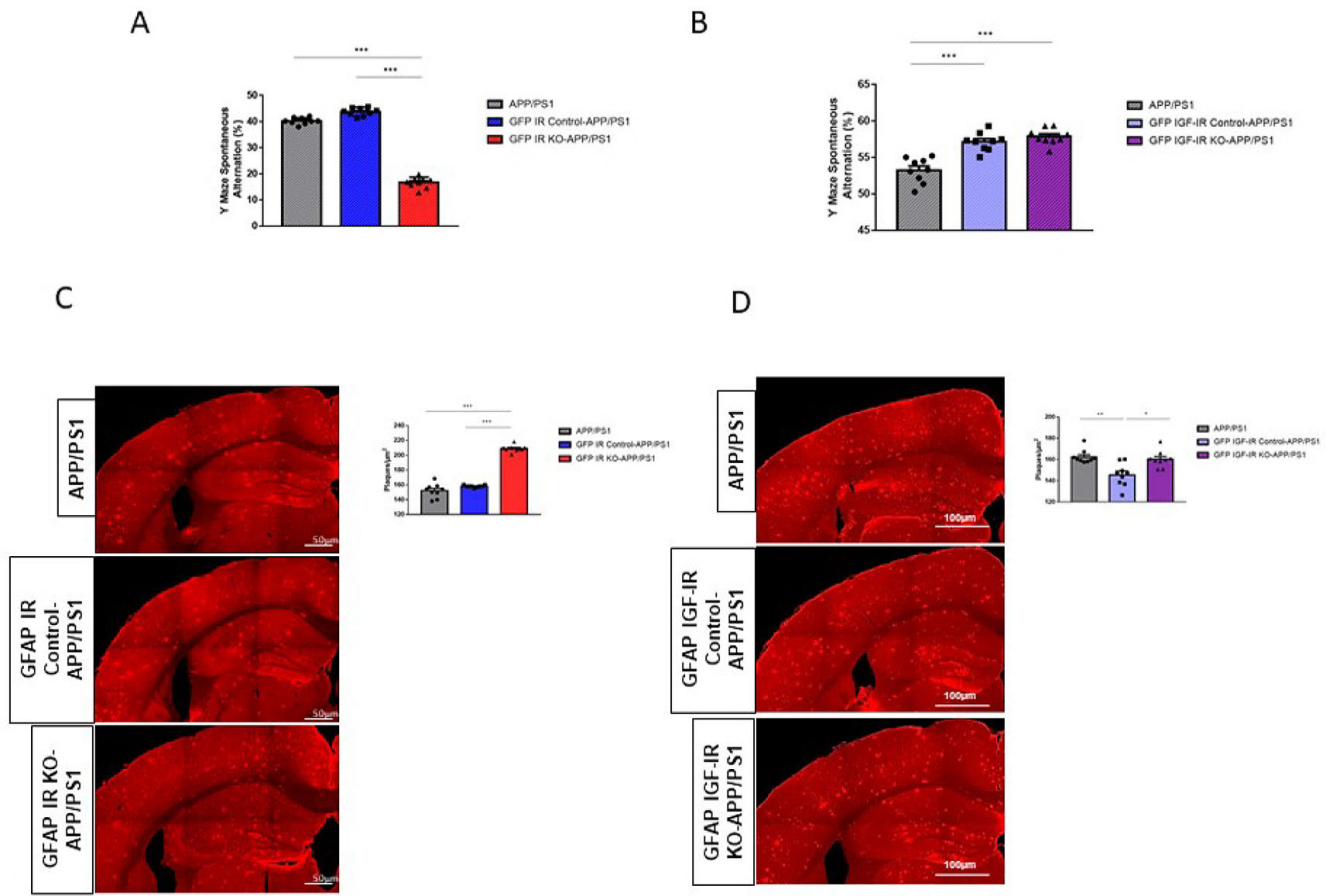
Modulation of Alzheimer´s-like pathology in GFAP IR KO/APP-PS1 and GFAP IGF-IR KO/APP-PS1 mice. A, Performance in the working memory version of the Y maze was impaired in GFAP IR KO APP/PS1 mice (n=8 per group; One-way ANOVA, F=73.23; ***p<0.001; Tukey’s Multiple comparison test, APP/PS1 vs. GFP IR KO-APP/PS1: ***p<0.001, GFP IR Control-APP/PS1 vs. GFP IR KO-APP/PS1: ***p<0.001). B, Working memory determined in the Y maze remained unaltered in GFAP IGF-IR KO APP/PS1 mice and controls (n=8 per group; One-way ANOVA, F=2.9; p=0.07). C, Amyloid plaques in the parietal cortex and hippocampus in GFAP IR KO/APP-PS1 mice and controls. Representative photomicrographs showing amyloid plaques (red). Histograms show number of plaques/μm ^2^ in three experimental groups (n=8 per group; One-way ANOVA, F=25.78; ***p<0.001; Tukey’s Multiple comparison test, APP/PS1 vs. GFP IR KO-APP/PS1: ***p<0.01, GFP IR Control-APP/PS1 vs. GFP IR KO-APP/PS1: ***p<0.001). D, Amyloid plaques in the parietal cortex and hippocampus in GFAP IGF-IR KO/APP-PS1 mice and controls does not show differences between groups. Representative photomicrographs showing amyloid plaques (red). Histograms show number of plaques/μm ^2^ in the three experimental groups (n=8 per group; One-way ANOVA, F=1.35; p=0.32). IR=insulin receptors, GFAP=glial fibrillary astrocytic protein.

Mice lacking IGF-IR in neurons show reduced AD-like pathology when cross-bred with a mouse AD model ( Gontier *et al*., 2015), whereas mice lacking IR in neurons have not shown changes in AD-like pathology ( Freude *et al*., 2009b). To analyze possible cell-dependent actions of these receptors in AD-like pathology, we crossed either GFAP IR KO or GFAP IGF-IR KO mice with APP/PS1 mice to obtain compound mutants and determined the impact of these receptors in memory loss associated to AD pathology seen in this mouse model. We observed that double GFAP IR KO/APP-PS1 mice presented significantly greater working memory loss compared to controls, as indicated by reduced spontaneous alternation in the Y maze ( Figure 5A). In contrast, double GFAP IGF-IR KO/APP-PS1 showed enhanced cognition when compared to APP/PS1 mice, but no changes when compared to vehicle-treated GFAP IGF-IR/APP-PS1 mice ( Figure 5B). Importantly, vehicle-treated control with preserved IGF-IR activity in astrocytes also showed enhanced cognition when compared to APP/PS1 mice ( Figure 5B). Associated to greater memory loss we observed greater amyloid load in GFAP IR KO/APP-PS1 mice ( Figure 5C), while in GFAP IGF-IR KO/APP-PS1 mice changes in amyloid plaque load were, again, control-dependent ( Figure 5D). When compared to APP/PS1 mice, no changes were seen, but when compared to vehicle-treated controls, plaque load was increased. Of note, vehicle-treated controls show reduced plaque load when compared to APP/PS1 controls ( Figure 5D).

## Discussion

The present results confirm and extend previous data of behavioral disturbances in GFAP IR KO or GFAP IGF-IR KO mice ( Cai *et al*., 2018; Noriega-Prieto *et al*., 2021), and point to cell, receptor and context-specific actions of these receptors in the brain. These observations also indicate that insulin and IGF-I receptors in astrocytes play different roles in regulating memory and plaque formation in response to AD-like familial amyloidosis.

Absence of astrocytic IR led to deteriorated performance in mood-related tests without affecting cognitive tests such as the Y and Barnes mazes. The latter agrees with no changes in cognitive performance in the absence of IR in neurons ( Plum *et al*., 2005), although more detailed studies are needed to determine the role of the neuronal IR in cognition, sociality and mood. Conversely, knock-down of IGF-IR in astrocytes affected performance in spatial memory tests and novelty-seeking such as the Barnes and Y mazes dependent on contextual clues, without affecting performance in the open field or elevated plus maze measuring mood traits. These mice showed normal working memory, though ( Noriega-Prieto *et al*., 2021). Intriguingly, absence of IGF-IR in neurons alters mood and social interactions, together with cognitive disturbances ( Zegarra-Valdivia *et al*., 2021; Fernandez de Sevilla *et al*., 2022). Finally, combined loss of IR and IGF-IR in all brain cells within specific regions results in both mood and cognitive disturbances ( Soto *et al*., 2019). Thus, cell-specific actions of IR and IGF-IR receptors on mood and behavior appear the norm.

Reported discrepancies on the role of ILP receptors in the brain most probably arise from the varied experimental approaches used. This is true for both physiological and pathological processes. When the role of IGF-IR in brain proteostasis was determined, evidence was obtained using an heterozygous constitutive, whole body IGF-IR KO mouse bred in an APP/PS1 background ( Cohen *et al*., 2009). This mouse showed reduced AD-related functional deficits but larger amyloid plaques. Additional confirmation of an involvement of IGF-IR in AD-like pathology was obtained using a homozygous neuronal-only tamoxifen-regulated IGF-IR KO mouse bred in an APP/PS1 background ( Gontier *et al*., 2015). However, in this mouse, amyloid plaques and AD-related neuroinflammation were diminished, in agreement with previous observations in a Cre-dependent homozygous neuronal-only IGF-IR KO mouse bred in a mutant APP background ( Freude *et al*., 2009b). This mouse also showed reduced amyloidosis and AD-related mortality, but no effects on other AD-related pathology were reported ( Freude *et al*., 2009b). No noticeable effect of the absence of IR in these mice was observed either ( Freude *et al*., 2009b).

Our observations reinforce the notion that modification of AD-like pathology after manipulation of IR or IGF-IR activity in brain cells is highly dependent on experimental conditions. Thus, we observed increased plaque abundance and worsened working memory using the Y maze in double mutant GFAP IR KO/APP-PS1. This observation allows us to consider that astrocyte IR plays a protective role against AD-like pathology. However, when using GFAP IGF-IR KO/APP-PS1 mice, the situation is more complex. Working memory in the Y maze is improved in both double mutant GFAP IGF-IR/APP-PS1 mice, regardless of whether the IGF-IR was deleted, as vehicle control littermates show a similar enhanced performance in the Y maze. Conversely, while GFAP IGF-IR KO mice did not show changes in plaque load when compared to APP/PS1 controls, GFAP IGF-IR mice treated with vehicle show decreased plaque load. Therefore, we can conclude that the actions of IR and IGF-IR are highly dependent on the experimental model used and that in the case of IGF-IR, the control littermate group show changes when compared to control APP/PS1 mice, which poses a cautionary note on the interpretation of results.

Other variables should also be accounted for when analyzing these results. For instance, peripheral and central metabolism affects brain function, and mice lacking IR ( Garcia-Caceres *et al.*, 2016; Fernandez de Sevilla *et al.*, 2022) or IGF-IR ( Hernandez-Garzon *et al.*, 2016) and in preparation) in astrocytes show disturbed blood glucose regulation in a time- and sex-dependent fashion. Disturbed brain function related to inflammation, oxidative stress ( Fernandez de Sevilla *et al.*, 2022) or apoptosis could also help explain the various phenotypes observed in mice lacking IR or IGF-IR in astrocytes. Underlying mechanisms will need to be studied in detail in future studies.

Several limitations should be stated. Although mouse models are successfully used to mimic human physiology and pathology, species-specific differences between mice and humans, should always be kept in mind when translating these observations. The reduced sample size in each experiment contributes to potential imprecision. Since bias in behavioral studies in experimental animals include sex of the experimenter performing the test, both male and female experimenters carried out these analyses. Together with the fact that mouse models of AD-like pathology, which are based in the genetic, least frequent type of AD, lack important aspects of the disease (most prominently, widespread neuronal loss), we consider that with the current available data, the role of ILP receptors in AD pathology remains undefined. Until better animal models of AD become available, and experimental approaches manipulating IR and IGF-IR activity are harmonized, we think this search should be re-formulated.

## Data availability

### Underlying data

Harvard Dataverse: DATA SET - ASTROCYTE INSULIN AND INSULIN-LIKE GROWTH FACTOR I (IGF-I) RECEPTORS. https://doi.org/10.7910/DVN/Y7K97E ( Zegarra-Valdivia *et al*., 2022).

This project contains the following underlying data:

- APP-PS1 (para FIRKOTAPP) 10x ProjMax001.tif
- APP-PS1(para BIRKOTAPP) 10x ProjMax001.tif
- BIRKOTAPP Control 10x ProjMax001.tif
- BIRKOTAPP KO 10x ProjMax001.tif
- DATA SET - ASTROCYTE INSULIN AND INSULIN-LIKE GROWTH FACTOR I (IGF-I) RECEPTORS v.2.xlsx
- FIRKOTAPP Control10x ProjMax001.tif
- FIRKOTAPP KO 10x ProjMax001.tif
- qPCR Data - Protocol.docx
- qPCR InsR-IGF1R Ct values.xlsx

## Reporting guidelines

Harvard Dataverse: ARRIVE checklist for ‘Insulin and insulin-like growth factor-I receptors in astrocytes exert different effects on behavior and Alzheimer’s-like pathology’. https://doi.org/10.7910/DVN/Y7K97E.

Data are available under the terms of the Creative Commons Zero “No rights reserved” data waiver (CC0 1.0 Public domain dedication).
