# Supplementary material for: Insulin and insulin-like growth factor-I receptors in astrocytes exert different effects on behavior and Alzheimer´s-like pathology
Source: F1000Res. 2022 Dec 20;11:663. Originally published 2022 Jun 16. [Version 3] doi: 10.12688/f1000research.121901.3 (PMC9823242; doi:10.12688/f1000research.121901.3)
Supplement: Peer review history

# Peer review: Reviewer response for version 3

**DOI:** 10.5256/f1000research.142193.r158472

**Published:** 2023-01-06

**Authors:** Susana Cardoso

**Affiliations:** 1 CNC-Center for Neuroscience and Cell Biology, CIBB-Center for Innovative Biomedicine and Biotechnology, University of Coimbra, Coimbra, Portugal

I have no further questions/comments.

Is the work clearly and accurately presented and does it cite the current literature?

Yes

If applicable, is the statistical analysis and its interpretation appropriate?

Partly

Are all the source data underlying the results available to ensure full reproducibility?

Yes

Is the study design appropriate and is the work technically sound?

Partly

Are the conclusions drawn adequately supported by the results?

Partly

Are sufficient details of methods and analysis provided to allow replication by others?

No

Reviewer Expertise:

Brain energy metabolism, Mitochondria, brain signaling pathways

I confirm that I have read this submission and believe that I have an appropriate level of expertise to confirm that it is of an acceptable scientific standard.

# Peer review: Reviewer response for version 2

**DOI:** 10.5256/f1000research.140877.r157009

**Published:** 2022-12-20

**Authors:** Andre Kleinridders

**Affiliations:** 1 Department of Molecular and Experimental Nutritional Medicine, Institute of Nutritional Science, University of Potsdam, Nuthetal, Germany

The authors have addressed the questions.

 One minor change is needed: In Figure 1C a T-test was used, but in Fig. 3D the correct 2Way ANOVA was used. Please change 1C accordingly.

 I have no further questions.

Is the work clearly and accurately presented and does it cite the current literature?

Yes

If applicable, is the statistical analysis and its interpretation appropriate?

Yes

Are all the source data underlying the results available to ensure full reproducibility?

No

Is the study design appropriate and is the work technically sound?

Yes

Are the conclusions drawn adequately supported by the results?

Yes

Are sufficient details of methods and analysis provided to allow replication by others?

No

Reviewer Expertise:

insulin signaling, brain metabolism, behavior, diabetes, obesity

I confirm that I have read this submission and believe that I have an appropriate level of expertise to confirm that it is of an acceptable scientific standard.

# Peer review: Reviewer response for version 2

**DOI:** 10.5256/f1000research.140877.r157010

**Published:** 2022-12-06

**Authors:** Susana Cardoso

**Affiliations:** 1 CNC-Center for Neuroscience and Cell Biology, CIBB-Center for Innovative Biomedicine and Biotechnology, University of Coimbra, Coimbra, Portugal

The authors have satisfactorily addressed the concerns and comments raised by the reviewers. 

 However, it was noticed that this revised version of the manuscript contains two new figures in the panel of Figure 4 (E and F) that are not mentioned in the paper and seem to be misplaced. Please check this. Further, if those are meant to be included, you will also need to (re)include the qPCR subheading in methods.

Is the work clearly and accurately presented and does it cite the current literature?

Yes

If applicable, is the statistical analysis and its interpretation appropriate?

Partly

Are all the source data underlying the results available to ensure full reproducibility?

Yes

Is the study design appropriate and is the work technically sound?

Partly

Are the conclusions drawn adequately supported by the results?

Partly

Are sufficient details of methods and analysis provided to allow replication by others?

No

Reviewer Expertise:

Brain energy metabolism, Mitochondria, brain signaling pathways

I confirm that I have read this submission and believe that I have an appropriate level of expertise to confirm that it is of an acceptable scientific standard, however I have significant reservations, as outlined above.

# Peer review: Reviewer response for version 1

**DOI:** 10.5256/f1000research.133816.r153403

**Published:** 2022-11-04

**Authors:** Susana Cardoso

**Affiliations:** 1 CNC-Center for Neuroscience and Cell Biology, CIBB-Center for Innovative Biomedicine and Biotechnology, University of Coimbra, Coimbra, Portugal

The manuscript by Zegarra-Valdivia and co-workers intended to elucidate the role of insulin receptors (IR) and insulin-growth factor 1 receptor (IGF-1R) in astrocytes. In particular, the authors had a specific interest in investigating the role of both receptors in mood and cognitive traits of young and older mice as well as in an AD-like context. To accomplish their goals, the authors used different KO models and performed the appropriate behavioral analysis.

 Overall, the rational of the study looks interesting, but the data presented seems to be quite preliminary to present conclusive findings.

 Please consider addressing the following points during the revision process:

1. Starting with the results, it is quite visible that the number of animals used to perform the forced swim test is not the same as the ones submitted to the other tests (Fig. 1). Do the authors have any justification for this? Such discrepancy can introduce significant bias on the data analysis.
2. Also, why is the number of animals used to evaluate cognitive behavior in IGF-1R KO (Fig.3) so different from the number of animals used to evaluate mood homeostasis (Fig.4)?
3. The graphs of Figure 5A and 5C do not have the individuals’ data points. Please check this.
4. Although the authors performed a reasonable detailed description of the methods, there are some points missing. It is mentioned that the authors used young and older mice of the IR KO genotype, but is not clear what was the age of the mice used from the IGF-1R genotype? And why were two age points for the IR-KO mice used and only one age point for the IGF-1R KO mice?
5. Also, evidence should be provided that the experimental mice are really KO in astrocytes as well as that the KO is not all-body.
6. In methods qPCR technique is described, but the manuscript does not have any mRNA data. Please check this.
7. Figure 5 should have data from control animals per se. The results presented in Figure 5B and 5D are quite difficult to understand. What are the genotypic differences between the APP/PS1 mice and the GFP-IGF-1R Control-APP/PS1 mice?

Is the work clearly and accurately presented and does it cite the current literature?

Yes

If applicable, is the statistical analysis and its interpretation appropriate?

Partly

Are all the source data underlying the results available to ensure full reproducibility?

Yes

Is the study design appropriate and is the work technically sound?

Partly

Are the conclusions drawn adequately supported by the results?

Partly

Are sufficient details of methods and analysis provided to allow replication by others?

No

Reviewer Expertise:

Brain energy metabolism, Mitochondria, brain signaling pathways

I confirm that I have read this submission and believe that I have an appropriate level of expertise to state that I do not consider it to be of an acceptable scientific standard, for reasons outlined above.

# Response

**Published:** 2022-11-07

**Authors:** Ignacio Torres Aleman

**Affiliations:** Achucarro Basque Center for Neuroscience, Spain

*"Starting with the results, it is quite visible that the number of animals used to perform the forced swim test is not the same as the ones submitted to the other tests (Fig. 1). Do the authors have any justification for this? Such discrepancy can introduce significant bias on the data analysis."*

**Answer:** Thank you for your comment. Depressive-like behavior in GFAP-IR KO mice was already reported by Cai et al (JCI 2018). These authors described the presence of mood alterations in young female GFAP-IR KO mice while males appear to have only minor deficits. The purpose of our study is to compare this model irrespective of sex differences in the context of AD as the role of IR in this disease is controversial. For this reason, we first confirmed that GFAP-IR KO mice (when both sexes are pooled together) show mood disturbances. In our hands, we found these disturbances in adult mice only (>6 months old) while Cai et al found them already in 4 month old mice, probably because we pooled together both sexes. Since we just wanted to confirm observations by others, we did not use more animals. In the rest of tests, no previous information was available, and for this reason we used more animals. In the case of the EPM test we used many more animals because Cai et al reported increased anxiety (without using the EPM test) in GFAP IR KO mice, but we did not see changes in this behavioral trait. 

*"Also, why is the number of animals used to evaluate cognitive behavior in IGF-1R KO (Fig.3) so different from the number of animals used to evaluate mood homeostasis (Fig.4)?"*

**Answer:** Figure 3: in cognitive tests we obtained significant differences using samples sizes of 8-12 animals/group. Since in GFAP-IR KO mice we had detected mood disturbances, we wanted to confirm that in GFAP IGF-IR KO mice mood is not altered (Figure 4). For this reason, we increased sample size to be sure that it was not a problem of sample size. It is quite intriguing that mice lacking IR in astrocytes show mood disturbances but intact cognition while mice lacking IGF-IR in astrocytes show altered cognition and intact mood.

*"The graphs of Figure 5A and 5C do not have the individuals’ data points. Please check this."*

**Answer:** Corrected. Apologies for the oversight.

*"Although the authors performed a reasonable detailed description of the methods, there are some points missing. It is mentioned that the authors used young and older mice of the IR KO genotype, but is not clear what was the age of the mice used from the IGF-1R genotype? And why were two age points for the IR-KO mice used and only one age point for the IGF-1R KO mice?"*

**Answer:** Young (< 6 months old) and adult (>6 months old) mice were used in both genotypes. This is now stated in the manuscript (Results, first subheading, second paragraph). The other part of this question has already been answered above.

*"Also, evidence should be provided that the experimental mice are really KO in astrocytes as well as that the KO is not all-body."*

**Answer**: We copy the answer already given to the other reviewer addressing this point: Both mouse models have been characterized by us in different publications: Garcia-Caceres et al Cell 2016, Hernandez-Garzon et al Glia 2016, and Noriega-Prieto J Neurosci 2021. Efficiency of Cre recombination after tamoxifen injection was shown in those publications. We did not examine all brain regions for deletion of IR or IGF-I respectively, only cortex and hippocampus. These two regions are involved in all tested behaviors. As indicated in the Glia 2016 publication, brain IR levels in IGF-IR KO mice were similar to wild types and these mice responded normally to insulin. Also brain IGF-IR levels in IR KOs were similar to wild type levels, and responded normal to IGF-I (Fernandez et al, PNAS 2022). We now address these points in the corrected manuscript, adding these supporting references.

*"In methods qPCR technique is described, but the manuscript does not have any mRNA data. Please check this."*

**Answer: **Thank you for the comment. In a draft version of the manuscript we were including mRNA levels in both genotypes, that is the reason for this error. We have deleted this subheading.

*"Figure 5 should have data from control animals per se. The results presented in Figure 5B and 5D are quite difficult to understand. What are the genotypic differences between the APP/PS1 mice and the GFP-IGF-1R Control-APP/PS1 mice?"*

**Answer:** Two types of controls are included in this Figure; APP/PS1 and APP/PS1x GFAP IGF-IR littermates. APP/PS1 mice have extensively been shown (including our previous publications) to present deficits in the Y maze and Abeta plaques. We agree that the results are quite difficult to explain, but they illustrate our main point: these mouse models are not adequate to ascribe a role for IGF-IR (or IR) in AD since the results are highly dependent on the model used. To our knowledge, previous results compared littermates and KOs in an APP/PS1 background, without including APP/PS1.

# Peer review: Reviewer response for version 1

**DOI:** 10.5256/f1000research.133816.r141517

**Published:** 2022-07-25

**Authors:** Andre Kleinridders

**Affiliations:** 1 Department of Molecular and Experimental Nutritional Medicine, Institute of Nutritional Science, University of Potsdam, Nuthetal, Germany

Zegarra-Valdivia et al., Insulin and insulin-like growth factor-I receptors in astrocytes exert different effects on behavior and Alzheimer´s-like pathology

 In their manuscript, Zegarra-Valdivia and colleagues investigate the effect of Insulin receptor (IR) and IGF-1 receptor (IGF-1R) deficiency in astrocytes on behavior using a tamoxifen-inducible knockout approach.

 The authors show that time-dependent IR deletion in GFAP astrocytes (GFAP IR KO mice) develop mood alterations, while cognition is not impacted. Yet, deficiency of IGF-1R in astrocytes causes a more severe behavioral phenotype with decreased cognition and depressive-like behavior. When these mouse models were bred with APP/PS1 mice, a model of familial Alzheimer´s disease (AD), the lack of IR worsened the phenotype, while deficiency of IGF-1R attenuated this phenotype. Yet, lox/lox animals showed the same phenomenon, as KO mice, questioning the effect of IGF-1R in this scenario. It remains elusive, why the observed phenotype is present, as no molecular insights were given.

 Overall the finding in this manuscript is very interesting and expands knowledge about the effect of insulin/IGF signaling in the brain on behavior. It addresses an important question, whether deletion of IR or IGF-1R in astrocytes impact behavior when deleted in adulthood. As most humans acquire insulin resistance while aging and not in their youth, these mouse models exhibit an important feature of age-associated brain insulin resistance and shed light into late-term complications. Thus the use of a tamoxifen-inducible knockout (KO) model is appropriate to gain more insights into time-dependent effects of IR and IGF-1R deficiency in astrocytes.

 Following issues with their presented data and conclusions should be addressed, to help the unfamiliar reader to better understand their results and compare it to already published literature.

1. The authors need to show tamoxifen-induced deletion deficiency, to better understand their acquired data. The behavioral traits, which have been investigated, are impacted by various brain regions. Is the IR or IGF-1R efficiently deleted in astrocytes in those regions? Is IR upregulated in IGF-1R KO mice and vice versa?
2. Does the lack of IR or IGF-1R alters blood glucose levels, as hyper- and hypoglycemia exhibit profound effects on brain physiology?
3. Do these mice exhibit neuroinflammation, oxidative stress or even apoptosis? Markers such as GFAP, Iba, lipid peroxidation etc. should be investigated to gain insights into potential mechanisms.
4. It has been shown that lack of IR or IGF-1R in astrocytes alters brain energy metabolism. Do mice with tamoxifen-induced IR and IGF-1R deletion in astrocytes show a similar effect?
5. Why does IR deficiency worsens the phenotype in APP/PS1 mice, yet IGF1R deletion not? What are proposed mechanisms?
6. Why do APP/PS1 lox/lox IGF-1R mice show a different phenotype compared to APP/PS1 mice? Why do these control mice exhibit the same phenotype as APP/PS1 IGF-1R KO animals? Here more research is needed.
7. Could the authors please state how many males and females participated for each experiment? Does ‘sex-balanced manner’ stand for an equal number of males and females and were there differences between the different tests? Is there a sex-specific difference, as it has been shown for conditional KO animals?

Minor comments:

1. In Figure 1A and B the authors show results of the Mousetail suspension test and Forced swimming test which were not described. The authors should add the test description in the method section.
2. Figure 5A and C do not show single data points compared to other figures. Please change accordingly.

Is the work clearly and accurately presented and does it cite the current literature?

Yes

If applicable, is the statistical analysis and its interpretation appropriate?

Yes

Are all the source data underlying the results available to ensure full reproducibility?

No

Is the study design appropriate and is the work technically sound?

Yes

Are the conclusions drawn adequately supported by the results?

Yes

Are sufficient details of methods and analysis provided to allow replication by others?

No

Reviewer Expertise:

insulin signaling, brain metabolism, behavior, diabetes, obesity

I confirm that I have read this submission and believe that I have an appropriate level of expertise to confirm that it is of an acceptable scientific standard, however I have significant reservations, as outlined above.

# Response

**Published:** 2022-11-07

**Authors:** Ignacio Torres Aleman

**Affiliations:** Achucarro Basque Center for Neuroscience, Spain

*"The authors need to show tamoxifen-induced deletion deficiency, to better understand their acquired data. The behavioral traits, which have been investigated, are impacted by various brain regions. Is the IR or IGF-1R efficiently deleted in astrocytes in those regions? Is IR upregulated in IGF-1R KO mice and vice versa?"*

**Answer:** Both mouse models have been described in detail by us in previous publications: Garcia-Caceres et al Cell 2016, Hernandez-Garzon et al Glia 2016, and Noriega-Prieto J Neurosci 2021. Efficiency of Cre recombination after tamoxifen injection was shown in those publications. We did not examine all brain regions for deletion of IR or IGF-I respectively, only cortex and hippocampus. These two regions are involved in all tested behaviors. As indicated in the Glia 2016 publication, brain IR levels in IGF-IR KO mice were similar to wild types and these mice responded normally to insulin. Conversely, brain IGF-IR levels in IR KOs were similar to wild type levels, and responded normal to IGF-I (Fernandez et al, PNAS 2022). We now address these points in the corrected manuscript, adding these supporting references.

*"Does the lack of IR or IGF-1R alters blood glucose levels, as hyper- and hypoglycemia exhibit profound effects on brain physiology?"*

**Answer:** Indeed, peripheral glucose levels will impact on brain physiology. The pattern of changes in blood glucose in these mice is time and sex-dependent. Young, but not middle-aged GFAP IR KO mice show hyperglycemia (Fernandez et al, PNAS 2022) and glucose intolerance (Garcia-Caceres et al, Cell 2016). Female, but not male GFAP-IGF-IR KO mice show glucose resistance (unpublished). We now introduce these data to nuance the discussion.

*"Do these mice exhibit neuroinflammation, oxidative stress or even apoptosis? Markers such as GFAP, Iba, lipid peroxidation etc. should be investigated to gain insights into potential mechanisms."*

**Answer:** Indeed, all these factors could help explain the observed phenotypes. We only have analyzed in greater detail GFAP-IR KO mice, that show oxidative stress (Fernandez et al PNAS 2022). We have included new comments in the discussion to address this point.

*"It has been shown that lack of IR or IGF-1R in astrocytes alters brain energy metabolism. Do mice with tamoxifen-induced IR and IGF-1R deletion in astrocytes show a similar effect?"*

**Answer:** Yes, both IR (Fernandez et al, PNAS 2022) and IGF-IR (Hernandez-Garzon et al, Glia 2016, and in preparation) show changes in brain glucose metabolism. This comment has also been introduced in the discussion. 

*"Why does IR deficiency worsens the phenotype in APP/PS1 mice, yet IGF1R deletion not?* *What are proposed mechanisms?"*

**Answer:** This is a key question. The observational data that we now present does not intend to answer it and will involve a variety of mechanistic studies. We wanted to publish these observations to illustrate that the effects of IR and IGF-IR in the APP/PS1 mouse depend on many variables not accounted for in previous studies. This was our solely aim. Analysis of underlying mechanisms in these specific models will in all probability unveil mechanisms related to these specific models. Our point is that with the current available approaches is premature to establish a role of IR and IGF-IR in Alzheimer disease.  

*"Why do APP/PS1 lox/lox IGF-1R mice show a different phenotype compared to APP/PS1 mice? Why do these control mice exhibit the same phenotype as APP/PS1 IGF-1R KO animals?* *Here more research is needed."*

**Answer:** Indeed, more research is needed if we want to answer these key points. We do not have any evidenced-based explanation. Floxing the IGF-IR gene may have unknown functional consequences. We believe that this type of observations are not enough to support a deleterious role of IGF-IR in Alzheimer disease, which has been until now widely held.

*"Could the authors please state how many males and females participated for each experiment? Does ‘sex-balanced manner’ stand for an equal number of males and females and were there differences between the different tests? Is there a sex-specific difference, as it has been shown for conditional KO animals?"*

**Answer: **Both sexes were used in similar proportions but not 50/50 in all experiments, in some 40/60 ratio was used. There may be sex differences (as seen in various traits in these KOs), but we did not consider necessary to use larger number of animals (also for ethical considerations) since our results already document that these models are not appropriate to reach robust conclusions about the role of IR/IGF-IR in mouse model of AD.

*Minor comments:*

1. *"In Figure 1A and B the authors show results of the Mouse tail suspension test and Forced swimming test which were not described. The authors should add the test description in the method section."*
  Corrected as indicated (see new methods). Apologies for the oversight.
2. *"Figure 5A and C do not show single data points compared to other figures. Please change accordingly."*
  Corrected as indicated. Again, apologies for the oversight.
